# Effects of supplementing bile acids on the production performance, fatty acid and bile acid composition, and gut microbiota in transition dairy cows

**DOI:** 10.1186/s40104-025-01207-8

**Published:** 2025-06-12

**Authors:** Lei Li, Jiaxiao Li, Zhihui Liu, Zihan Jin, Mengyang Wang, Ying Wu, Zhihong Zhang, Xinfeng Hou, Junhu Yao, Jun Zhang

**Affiliations:** 1 College of Animal Science and Technology, Northwest A&F University, Yangling, 712100 China; 2 National Center of Technology Innovation for Dairy, Inner Mongolia Dairy Technology Research Institute Co. Ltd, Hohhot, 010100 China; 3 Hebei Leyuan Animal Husbandry Co., Ltd, Shijiazhuang, 050000 China

**Keywords:** Bile acids, Fatty acids, Gut microbiota, Production performance, Transition dairy cow

## Abstract

**Background:**

During the transition period, cows are prone to negative energy balance, which can lead to a decline in production performance and health in severe cases. In recent years, it has been discovered that bile acids (BAs) can act not only as fat emulsifiers but also as signaling molecules to regulate body metabolism. Although BAs have been used to some extent in monogastric and aquatic animals, their role in ruminants, particularly in transition cows, remains unclear. Therefore, this study aimed to determine the effects of BAs on the production performance, milk and plasma fatty acid and BA composition, and fecal microbiota in transition dairy cows.

**Results:**

Forty-six healthy transition Holstein dairy cows with similar conditions were randomly divided into two groups and supplemented with 0 or 20 g/d of BAs from 21 d before the expected calving to 21 d after calving. The production performance was tracked until 60 d after calving. The results indicated that BA supplementation significantly improved postpartum milk fat content and yields as well as the yields of unsaturated fatty acids, monounsaturated fatty acids, and polyunsaturated fatty acids in milk. There was a significant increase in the concentration of triglyceride and the proportion of C ≤ 16 fatty acids in the plasma of cows supplemented with BAs, while the concentration of β-hydroxybutyrate and the proportion of C > 16 fatty acids in the plasma decreased significantly. BA supplementation significantly altered the composition of the fecal bacterial community and increased the relative abundance of bacteria beneficial for BA metabolism and transformation (*Romboutsia*, *Clostridium sensu_stricto_6*, and *Clostridium sensu_stricto_1*). Functional prediction analysis showed that the relative abundance of bile salt hydrolase, 7α-hydroxysteroid dehydrogenase, and BA inducible E as well as the pathways related to BA metabolism also significantly increased in cows supplemented BAs. In addition, BA supplementation significantly altered the composition of plasma and fecal BAs, particularly increasing circulating secondary BA concentration, which might induce the complete oxidation of fatty acids in the liver and further reduce the concentration of β-hydroxybutyrate.

**Conclusions:**

These findings highlight the potential benefits of BA supplementation in improving milk yields and quality, as well as influencing metabolic pathways in transition dairy cows. Meanwhile, further studies are needed to elucidate the underlying mechanisms and explore the broader implications of these results by using more tissue samples.

**Supplementary Information:**

The online version contains supplementary material available at 10.1186/s40104-025-01207-8.

## Background

The transition period is considered the most challenging time in a dairy cow’s production cycle [1]. During this period, cows’ lactation energy requirements increase dramatically, while the feed intake is low and increases slowly, resulting in a state of negative energy balance (NEB). When the cow is in a severe NEB situation, a large amount of nonesterified fatty acids (NEFAs) released from the adipose tissue will go into the liver to produce energy to meet the postpartum lactation demand as well as be converted into ketone bodies or be re-esterified to triglyceride (TG) [2–5]. Thus, dairy cows in the early lactation stage are prone to ketosis (hyperketonemia) and fatty liver, which reduce production performance and impair health status [6, 7]. To some extent, these metabolic diseases are primarily caused by the dysregulation of lipid and glucose metabolism during the transition period [5, 8, 9]. 

Milk fat is one of the main nutritional components for offspring and is also one of the most complex lipids known in terms of composition and structure, which is directly related to the economic benefits of dairy farms and the healthy growth of calves. As the main component of milk fat, TG is synthesized in mammary epithelial cells from α-phosphoglycerides and fatty acids (FAs) and accounting for approximately 95% in milk fat [10]. In dairy cows, there are two pathways for the synthesis of FAs in milk, namely the de novo synthesized pathway in mammary gland epithelial cells and the direct uptake of FAs from the blood [11]. Thus, FAs exhibit a wide range of physiological activities and biological functions [12, 13]. However, disordered glucose and lipid metabolism can also negatively affect milk FA synthesis. Research has shown that the ability of cows to manage energy intake and demand during the transition period is one of the important factors determining the success or failure of later lactation performance [14]. Only cows who can successfully adapt to the onset of lactation and pass through the NEB period will have high production performance and health status for the entire lactation period [6].

Bile acids (BAs) are specific products of cholesterol catabolism in the liver and are a major component of human and animal bile [15]. There is growing evidence demonstrating that BAs not only act as emulsifiers and promoters of lipid digestion and absorption in the small intestine but also can mediate host glucose and lipid metabolism by binding to their receptors and their downstream intermediates [16–18]. After being synthesized in the hepatocytes and bound to glycine or taurine, primary BA (PBA) is released into the gut and then converted into secondary BA (SBA) by the modifications of gut microbes. When BAs reach the distal small intestine, only a small portion of BAs pass through the colon and are excreted in the feces. Most of the BAs are reabsorbed by the intestinal epithelium and returned to the liver through the portal vein, which is called the enterohepatic circulation of BAs [19]. Thus, BAs have close interactions with gut microbes [20]. The reabsorbed BAs also play regulatory roles in lipid metabolism in the liver [21].

The interactions among gut microbiota, BAs, and host metabolism have been reported in previous studies. Firstly, as a natural antimicrobial compound, BAs can affect the host's physiological status through the selective remodeling of gut flora composition [22–24]. Secondly, changes in the abundance and composition of gut microorganisms due to physiological states and diets can also affect the amount and composition of BAs, which in turn play an important role in regulating host cholesterol, TG, glucose levels, and insulin sensitivity [25–27]. For example, PBAs such as chenodeoxycholic acid can be converted by microbial 7α-hydroxysteroid dehydrogenase (7α-HSDH) into SBAs such as deoxycholic acid, which can maintain glucose and lipid homeostasis by activating BA receptors, thereby leading to the alleviation of metabolic syndrome [28, 29]. Due to these benefits, BAs have been used as a potential additive in pigs, poultry, and aquatic animals. However, the effects of BA supplementation on ruminants, especially on transition dairy cows, and the specific mechanisms of action are not fully understood. Therefore, this study aimed to investigate the effects of BA supplementation on production performance, milk and plasma fatty acid composition, BA metabolism, and fecal microbiota in postpartum dairy cows. These results will contribute to a better understanding of how BA supplementation affects the production performance and metabolic health of postpartum dairy cows, which will further provide new insights into the nutritional regulation and management strategies in transition cows.

## Materials and methods

This study was conducted in strict accordance with the guidelines of the Administration of Affairs Concerning Experimental Animals (Ministry of Science and Technology, China, revised 2004), and all the procedures were approved by the Institutional Animal Care and Use Committee of the Northwest A&F University (protocol number DK2021028).

### Animals, diets, and experimental design

Forty-six healthy multiparous Chinese Holstein dairy cows with similar parity (2.5 ± 0.51), body weight (863 ± 73 kg), body condition score (3.4 ± 0.10), due date, and previous milk yields (10,603 ± 2,533 kg) were selected from a large cohort of 5,000 cows from the First Ranch in Wei County (Xingtai City, Hebei Province, China) at the time when they arrived at the transition barn (about 22 d before calving). Cows were assigned into 2 groups using a completely randomized block design: (1) control group (CON, basal diet; *n* = 23) and (2) supplemented with 20 g/d BA product (BAS; *n* = 23). The BA product was a mixture consisting of chenodeoxycholic acid (18.6%) and hyodeoxycholic acid and hyocholic acid (combined 78.2%), which was the same as that used in previous studies [30, 31]. The dosage of BAs was determined following the manufacturer’s recommendation and a previous study in mid-lactation dairy cows [32]. Before every morning feeding, the 20-g BA product was mixed well with approximate 500 mL purified water and then administered into the esophagus through a homemade tube. The homemade tube was mainly connected by a hose between the funnel and a plastic hard tube with about 50 cm long and 5 cm in diameter. To ensure completely consumption of the BAs supplemented, the tube was rinsed again with another 300 mL purified water. Throughout the experimental period, cows were housed in a free-stall barn and had free access to water. A total mixed ration (TMR) was provided daily at 07:00 and 13:00 h in both prepartum and postpartum (Table S1). After calving, cows were milked four times daily at 02:00, 07:00, 13:00, and 21:00 h. The BA supplementation was started at 21 d before calving and stopped at 21 d after calving, and cows were observed until 60 d after calving.

### TMR and fecal sample collection and analysis

During the experiment, fresh and refused TMR samples were collected once a week and stored at −20 °C. At the end of the experiment, all the TMR samples were well mixed and subsampled, and then dried in an oven at 65 °C for 72 h and crushed using a grinder (FW100, Tianjin Taist Instrument Co., Ltd., Tianjin, China). The dietary nutrient composition such as dry matter (DM; method 930.15), crude protein (CP; method 976.05), ether extract (EE; method 920.39), Ash (method 955.03), calcium (Ca; method 985.35), and phosphorus (P; method 986.24) contents were analyzed according to the Association of Official Analytical Chemists International [33]. The neutral detergent fiber (NDF) and acid detergent fiber (ADF) contents were measured with heat-stable amylase and sodium sulfite using a fiber analyzer (A200i, ANKOM, NY, USA) according to previous methods [33, 34]. The dietary starch content was measured with polarimetry using a previous method [34].

Fecal samples were collected from the rectum of cows using a sterile swab (FS916, Swwip, Shenzhen Cleanmo Technology Co., Ltd., Shenzhen, China) before morning feeding on d 21 after calving and were immediately stored in liquid nitrogen for omics analysis.

### Rumen fluid collection and analysis

Before morning feeding on d 14 after calving, rumen fluid was obtained using an oral stomach tube, as described in a previous study [35]. The first 50 mL of rumen fluid was discarded to minimize saliva contamination. Two milliliters of rumen sample (solid and liquid fractions) was immediately frozen in liquid nitrogen and stored at −80 °C to minimize any possible microbial activities for later DNA extraction. Rumen pH was immediately measured after collection using a mobile pH meter (Starter 300; Ohaus Instruments Co. Ltd., Shanghai, China). After pH measurement, samples were passed through four layers of sterile cheesecloth and kept on ice until further processing. Filtered rumen fluid was centrifuged (17,000 × *g* for 30 min at 4 °C) to obtain a clear supernatant, which was further analyzed for NH_3_-N using a phenol-hypochlorite assay [36]. The concentrations of volatile fatty acids were measured with a gas chromatograph (GC) according to a previous report [37].

### Blood sample collection and analysis

Before morning feeding on d 21 after calving, blood samples were collected into 10-mL heparin-containing tubes (Jiangsu Kangjie Medical Equipment Co., Ltd., Taizhou, China) through venipuncture of the tailbone blood vessels just after fecal sampling and before rumen fluid sampling. The blood samples were immediately placed on ice after sampling and then centrifuged at 3,500 × *g* for 15 min to obtain plasma. Equivalent plasma samples were transferred into 2-mL tubes and stored at −20 °C and liquid nitrogen, respectively. The plasma individual metabolites, such as: glucose (GLU, cat#702029; Shandong Boke Biological Industry Co., Ltd., China), high-density lipoprotein (HDL, cat#702134), low-density lipoprotein (LDL, cat#702135), TG (cat#702133), total cholesterol (TC, cat#702132), total protein (TP, cat#702012), albumin (ALB, cat#702013), total BA (TBA, cat#702017), NEFA (cat#702110), β-hydroxybutyric acid (BHBA, cat#702059), and total bilirubin (TBIL, cat#702082) concentrations and alkaline phosphatase (ALP, cat#702136), alanine aminotransferase (ALT, cat#702011), and aspartate transaminase (AST, cat#702138) activity were analyzed by using a fully automated biochemical analyzer (BK-400, Shandong Boke Biological Industry Co., Ltd.) following the manufacturer’s instructions. Globulin (GLB) concentration was obtained based on the difference between TP and ALB concentrations. Plasma insulin (INS) concentration was determined using an insulin radioimmunoassay kit (XH6080, Beijing North Institute of Biological Technology, Beijing, China) following the manufacturer’s instructions, and the revised quantitative insulin sensitivity check index (RQUICKI) was calculated according to a previous report [38].

The FA composition in plasma samples was analyzed using GC as described by a previous study [39]. In brief, FAs were extracted from plasma using n-hexane/isopropanol, added to internal standard C19:0, and dried with nitrogen. Afterward, it was dissolved in n-hexane/methanol; alkaline esterified with potassium hydroxide methanol solution and acid esterified by hydrochloric acid methanol solution. After cooling, water and n-hexane were added to the mixture, and the supernatant was taken to the mark and dried over anhydrous Na_2_SO_4_. The measurement was conducted by an Agilent 7890N GC (Agilent, USA) equipped with HP-88 chromatographic column (100 m × 0.25 mm × 0.2 μm), and under specific conditions including maintaining at 120 °C for 10 min as the initial temperature, raising 230 °C at a rate of 3.2 °C/min and holding for 35 min, injection at 250 °C, and detection at 280 °C with 37 mixed FA methyl esters (Sigma, USA) as standards. All FA composition results were expressed in g/100 g total FAs.

### Milk sample collection and analysis

The milk yields were recorded daily using the ALPROTM system (DeLaval, Tumba, Sweden). After calving, milk samples were collected on d 12–13, 20–21, and 60–61. During each milking, milk samples were collected by a continuous milk sampling device (Tumba, Sweden) and then pooled in a proportion of 1:1:1:1 to obtain one daily sample after a day and night sampling. A portion mixture was stored at 4 °C for milk composition analysis, and another was stored at −20 °C for milk FAs analysis. Energy-corrected milk (ECM) was calculated as follows: (12.95 × fat yield) + (7.65 × protein yield) + (0.327 × milk yield) [40], and 4% fat-corrected milk (FCM) was calculated as follows: (0.4 × milk yield) + (15 × fat yield) [41]. The milk FA and plasma FA composition measurements were under the same GC system and condition but with different pre-treatment processes [42]. In short, the milk samples were methylated with 4 mL of 0.5 mol/L NaOH/methanol at 50 °C for 15 min, then methylated with 4 mL of 5% HCl/methanol at 50 °C for 1 h. After extracting 2 mL of n-hexane, the sample was conducted using an Agilent 7890N gas chromatograph system described above. All FA composition results were expressed as g/100 g of total FAs and g/d.

### DNA extraction and 16S rRNA gene sequencing

Microbial DNA of rumen and fecal samples was extracted from 18 randomly selected cows (*n* = 9 in each group) by using the E.Z.N.A. DNA kit (Omega Biotek, Norcross, GA, USA) according to the manufacturer’s protocol. The DNA concentration was measured with a Nanodrop-2000 (Thermo Fisher Scientific, Wilmington, DE, USA) and the quality was assessed using 1% agarose gel electrophoresis. Bacterial 16S rRNA gene fragments (V3–V4) in the extracted DNA were amplified using the forward primer 27F (5'-AGRGTTYGATYMTGGCTCAG-3'), and reverse primer 1492R (5'-RGYCCTTTGTTACGACTT-3') [43]. PCR products were visualized on 2% agarose gels and purified using the QIAquick gel extraction kit (Qiagen, Dusseldorf, Germany). Sequencing was done on an Illumina MiSeq PE300 paired-end platform by Shanghai Meiji Biomedical Technology Co., Ltd. (Shanghai, China). The sequences were analyzed using QIIME 2 (https://qiime2.org) with default parameters [44]. The amplicon sequence variants (ASV) were annotated according to the Silva bacteria database. The alpha diversity indices, including ACE, Chao1, Shannon, and Simpson, were calculated using QIIME2 with default parameters. The principal coordinates analysis (PCoA) was conducted based on the Bray Curtis distance algorithm to test the differences in microbial community structure composition between treatments, and analysis of similarities (ANOSIM) at the phylum level with 999 permutations was used to determine statistical significance. Phylogenetic investigation of communities was analyzed by PICRUSt2 (https://github.com/picrust/picrust2) with default parameters and gene banks such as the Kyoto Encyclopedia of Genes and Genomes (KEGG) [45].

The relative expression abundance of BA converting enzymes such as 7α-HSDH (EC1.1.1.159), BSH (EC3.5.1.24), and BaiE (EC3.5.1.59) were calculated based on microbial functional analysis using PICRUSt2 and KEGG functional abundance statistics and were expressed as the percentage of the total sequences, as described in one of our previous studies [31].

### Target metabolomic analysis of BAs in plasma and feces

Target metabolomes of plasma and fecal BAs were measured by a UHPLC-parallel reaction monitoring-MS method by Shanghai Biotree Biotech Co., Ltd. (Shanghai, China) as described in one of our previous studies [20]. Briefly, the samples (approximately 100 mg) were extracted with 1 mL of methanol using ultrasonic assistance. The resulting methanol extracts were centrifuged, filtered, and quantified using the UPLC-MS/MS system according to the established protocols [20, 46]. The UHPLC separation was performed using a UHPLC System (Vanquish, Thermo Fisher Scientific, San Jose, CA, USA) equipped with a UPLC BEH C18 column (150 mm × 2.1 mm, 1.7 μm, Waters). The mobile phase A was 1 mmol/L ammonium acetate and 0.1% acetic acid in the water, and the mobile phase B was acetonitrile. The column temperature was set at 50 °C. The auto-sampler temperature was set at 4 °C, and the injection volume was 1 μL. The standard curves were built by using the peak areas ratio for the analyte: the internal standard is *y*, and the concentration of the analyte (nmol/L) or (nmol/kg) is *x*. The least squares method was used for the regression fitting in Excel. The optimal accuracy and correlation coefficient (*R*^2^) are obtained using 1/*x* as weight.

### Statistical analysis

The animal performance data were first checked for normality and outliers using the UNIVARIATE procedure of SAS (version 9.4, SAS Institute Inc., Cary, NC, USA). The data for rumen fermentation parameters, plasma individual metabolites, milk yield and composition on the 9th week postpartum, milk FA composition and yield on d 21 postpartum, plasma FA composition, and plasma and fecal BA composition were analyzed using the PROC GLM procedure of SAS. The data for milk yield, milk composition, and milk composition yield during 1–3 weeks postpartum were analyzed using the PROC MIXED procedure with repeated measuring including treatment group, block (previous milk yield and due date), time (week or day), and their interaction as fixed effects, and cow as a random effect.

For the microbial data, the linear discriminant analysis (LDA) effect size (LEfSe) analysis (http://huttenhower.sph.harvard.edu/LEfSe) was used to identify bacterial groups with significant differences in relative abundance from domain to genus levels between two groups (LDA > 3.00, *P* < 0.05).

Fourteen cows were excluded from the sampling due to the following reasons: five cows with early birth, two cows with dystocia, one cow with miscarriage, two cows with ketosis, and four cows with milk fever. As a result, the CON and BAS groups had 15 and 17 cows at the end of the experiment, respectively. Results were reported as least squares mean. Statistical differences were declared at *P* < 0.05, and a tendency toward significance was considered at 0.05 ≤ *P* < 0.10.

## Results

### Effects of BA supplementation on rumen fermentation parameters and bacterial community in postpartum dairy cows

No significant differences in rumen fermentation parameters were found between the two groups on d 14 postpartum (Table S2). The BAS group had a greater Simpson value (*P* = 0.04) than the CON group (Fig. S1A). The PCoA and ANOSIM (*R* = 0.08, *P* = 0.14) showed no significant differences in the beta diversity between the two groups (Fig. S1B). A total of 16 phyla and 190 genera were detected in the rumen of the two groups. Firmicutes and Bacteroidota were the two most abundant phyla, accounting for 67.90% ± 0.01% and 26.70% ± 0.02% of the total sequences, respectively (Fig. S1C). *Lachnospiraceae NK3A20 group* and *Prevotella* were the two most abundant genera, accounting for 15.30% ± 0.02% and 14.80% ± 0.03% of the total sequences, respectively (Fig. S1D).

### Effects of BA supplementation on plasma individual metabolites and FA profiles in postpartum dairy cows

The BAS group had greater plasma TG (*P* < 0.01) concentration and ALT activity (*P* = 0.04) than the CON group on d 21 postpartum, while the BHBA concentration was lower (*P* = 0.04) in the BAS group (Table 1). The proportions of C12:0 (*P* = 0.03), C14:0 (*P* = 0.03), C16:0 (*P* = 0.02), were greater in the BAS group than in the CON group (Table 2), and the proportions of saturated FAs (*P* = 0.03) and C ≤ 16 (*P* = 0.02) were greater in the BAS group than in the CON group. On the contrary, the proportion of *cis*-5,8,11,14,17 C20:5 (*P* = 0.05) were lower in the BAS group. Similarly, the proportions of unsaturated FAs (*P* = 0.03) and C > 16 (*P* = 0.02) were lower in the BAS group. No significant differences were found in other plasma individual metabolites such as plasma NEFA concentration and FA profiles between these two groups.

**Table 1:** Effects of supplementing bile acids on plasma individual metabolites on d 21 postpartum in transition dairy cows

Items^1^	Treatments		SEM	*P*-value
	CON	BAS		
Energy metabolism				
GLU, mmol/L	2.42	2.63	0.125	0.43
BHBA, mmol/L	1.13	0.83	0.073	0.04
INS, μIU/mL	12.47	12.95	0.502	0.64
RQUICKI	0.40	0.39	0.004	0.52
Lipid metabolism				
TG, mmol/L	0.14	0.30	0.030	< 0.01
TC, mmol/L	3.50	3.74	0.145	0.42
TBA, μmol/L	170.22	137.01	11.914	0.17
TBIL, μmol/L	2.18	1.74	0.200	0.29
NEFA, mmol/L	0.64	0.71	0.044	0.42
Liver function markers				
TP, g/L	77.57	77.01	0.799	0.74
ALB, g/L	32.61	32.20	0.282	0.48
GLB, g/L	44.44	44.82	0.716	0.80
A/G	0.74	0.73	0.013	0.64
HDL, mmol/L	1.86	2.08	0.076	0.14
LDL, mmol/L	2.01	2.15	0.095	0.47
ALP, U/L	1.91	1.71	0.129	0.47
ALT, U/L	14.64	16.00	0.334	0.04
AST, U/L	123.86	119.10	3.912	0.55

^1^*GLU* Glucose, *BHBA* β-Hydroxybutyrate, *INS* Insulin, *RQUICKI* Revised quantitative insulin sensitivity check index, *TG* Triglyceride, *TC* Total cholesterol, *TBA* Total BA, *TBIL* Total bilirubin, *NEFA* Non-esterified fatty acid, *TP* Total protein, *ALB* Albumin, *GLB* Globulin, *A/G* Albumin: globulin ratio, *HDL* High-density lipoprotein, *LDL* Low-density lipoprotein, *ALP* Alkaline phosphatase, *ALT* Alanine aminotransferase, *AST* Aspartate transaminase. *SEM* Standard error of means. CON (*n* = 15) and BAS (*n* = 17), without and with supplementing 20 g/d of bile acids, respectively

**Table 2:** Effects of supplementing bile acids on plasma fatty acid composition on d 21 postpartum in transition dairy cows

Items, g/100 g of total FAs^1^	Treatments		SEM	*P*-value
	CON	BAS		
C4:0	0.24	0.12	0.029	0.03
C6:0	0.09	0.04	0.012	0.04
C8:0	0.05	0.04	0.019	0.87
C10:0	0.76	1.33	0.240	0.25
C11:0	0.25	0.23	0.025	0.72
C12:0	1.54	3.77	0.520	0.03
C13:0	0.19	0.14	0.028	0.42
C14:0	6.32	12.78	1.515	0.03
C14:1	0.27	0.27	0.033	0.96
C15:0	0.94	1.32	0.109	0.08
*cis*-10 C15:1	0.20	0.32	0.065	0.38
C16:0	22.75	26.24	0.806	0.02
*cis*-9 C16:1	0.52	0.45	0.074	0.63
C17:0	1.13	1.13	0.077	0.98
C17:1	2.96	2.12	0.378	0.28
C18:0	32.00	25.69	1.939	0.11
*cis*-9 C18:1	0.73	1.24	0.131	0.04
*trans*-9 C18:1	7.16	5.87	1.900	0.74
*trans*-9,12 C18:2	0.15	0.14	0.015	0.77
*cis*-9,12 C18:2	0.16	0.16	0.017	0.96
C20:0	0.14	0.16	0.034	0.79
*cis*-6,9,12 C18:3	0.42	0.41	0.062	0.98
*cis*-11 C20:1	5.37	4.39	0.496	0.33
*cis*-9,12,15 C18:3	0.22	0.31	0.028	0.09
C21:0	0.24	0.26	0.038	0.76
*cis*-11,14 C20:2	0.09	0.07	0.007	0.16
C22:0	0.27	0.22	0.039	0.57
*cis*-8,11,14 C20:3	2.86	2.30	0.348	0.43
*cis*-13 C22:1	0.65	0.50	0.070	0.27
*cis*-11,14,17 C20:3	5.44	3.92	0.459	0.10
*cis*-5,8,11,14 C20:4	3.58	2.50	0.405	0.22
C23:0	0.49	0.19	0.094	0.17
*cis*-13,16 C22:2	0.24	0.09	0.035	0.07
C24:0	0.89	0.71	0.098	0.37
*cis*-5,8,11,14,17 C20:5	0.24	0.13	0.029	0.05
*cis*-15 C24:1	0.17	0.12	0.018	0.23
*cis*-4,7,10,13,16,19 C22:6	0.28	0.32	0.074	0.81
SFAs	68.29	74.38	1.459	0.03
UFAs	31.71	25.62	1.459	0.03
MUFAs	18.04	15.27	1.493	0.36
PUFAs	13.67	10.35	1.122	0.14
C ≤ 16	33.61	46.60	2.832	0.02
C > 16	66.39	53.40	2.832	0.02

^1^*SFAs* Saturated fatty acids, *UFAs* Unsaturated fatty acids, *MUFAs* Monounsaturated fatty acids, *PUFAs* Polyunsaturated fatty acids. *SEM* Standard error of means. CON (*n* = 15) and BAS (*n* = 17), without and with supplementing 20 g/d of bile acids, respectively

### Effects of BA supplementation on milk yields, composition, and FA profiles in postpartum dairy cows

Even though milk yields was similar between these two groups, the BAS group had greater 4% FCM (*P* = 0.01) and ECM (*P* = 0.02) yields than the CON group during the first three weeks of lactation (Table 3). The milk fat content and yields were also greater in the BAS group than in the CON group during the first three weeks of lactation (*P* < 0.01), but no significant differences were found in the contents and yields of milk protein and lactose. In addition, no significant differences were found in milk composition and yields on the 9th week postpartum.

**Table 3:** Effects of supplementing bile acids on milk yields and composition at postpartum in transition dairy cows

Items^1^	Treatments		SEM	*P*-value		
	CON	BAS		Treatment	Time	Treatment × Time
Milk yield, kg/d						
Weeks 1–3	41.67	42.75	0.937	0.57	< 0.01	0.01
Weeks 4–9	53.53	52.78	0.591	0.53	< 0.01	0.71
4% FCM, kg/d						
Weeks 1–3	46.79	52.82	1.203	0.01	0.06	0.11
Week 9	53.70	52.91	1.423	0.79		
ECM, kg/d						
Weeks 1–3	50.19	55.81	1.226	0.02	0.08	0.11
Week 9	58.72	57.65	1.438	0.72		
Fat, %						
Weeks 1–3	4.25	4.92	0.08	< 0.01	0.65	0.53
Week 9	3.50	3.73	0.155	0.32		
Protein, %						
Weeks 1–3	3.27	3.31	0.017	0.26	< 0.01	0.20
Week 9	3.20	3.23	0.099	0.53		
Lactose, %						
Weeks 1–3	4.70	4.72	0.024	0.74	0.82	1.00
Week 9	4.67	4.70	0.145	0.72		
Fat, kg/d						
Weeks 1–3	1.92	2.28	0.057	< 0.01	0.08	0.14
Week 9	2.03	2.04	0.071	0.93		
Protein, kg/d						
Weeks 1–3	1.47	1.54	0.031	0.34	0.18	0.14
Week 9	1.86	1.80	0.045	0.52		
Lactose, kg/d						
Weeks 1–3	2.12	2.19	0.047	0.45	0.12	0.15
Week 9	2.72	2.62	0.065	0.48		

^1^*SEM* Standard error of means. CON (*n* = 15) and BAS (*n* = 17), without and with supplementing 20 g/d of bile acids, respectively

Even though proportions of C ≤ 16 and C > 16 FAs in milk were similar between these two groups (Table 4), the BAS group had lower proportions of *cis*-11,14 C20:2 (*P* = 0.02), *cis*-5,8,11,14,17 C20:5 (*P* = 0.04), and *cis*-15 C24:1 (*P* = 0.04) than the CON group. In terms of FA sources, the de novo, mixed, and preformed FAs were similar between the two groups. The yields of medium/long-chain FAs, including C8:0 (*P* < 0.01), C12:0 (*P* < 0.01), C14:0 (*P* < 0.01), C16:0 (*P* < 0.01), and *cis*-9 C16:1 (*P* < 0.01), was greater in the BAS group than in the CON group (Table 5). The yields of long-chain FAs, including C17:0 (*P* < 0.01), C17:1 (*P* = 0.03), *cis*-9 C18:1 (*P* < 0.01), and *cis*-9,12 C18:2 (*P* = 0.01), was also greater in the BAS group than in the CON group. The BAS group had greater unsaturated FAs (*P* < 0.01), monounsaturated FAs (*P* < 0.01), polyunsaturated FAs (*P* = 0.01), C ≤ 16 (*P* < 0.01), and C > 16 (*P* < 0.01) yields than the CON group, and the de novo, mixed, and preformed FAs yields was also greater in the BAS group than in the CON group (*P* < 0.01).

**Table 4:** Effects of supplementing bile acids on milk fatty acid composition at d 21 postpartum in transition dairy cows

Items, g/100 g of total FAs^1^	Treatments		SEM	*P*-value
	CON	BAS		
C4:0	1.69	1.68	0.033	0.22
C6:0	1.04	1.07	0.043	0.71
C8:0	0.69	0.77	0.039	0.36
C10:0	1.68	1.66	0.123	0.95
C11:0	0.07	0.05	0.005	0.20
C12:0	2.18	2.31	0.100	0.55
C13:0	0.10	0.09	0.006	0.57
C14:0	10.11	9.98	0.188	0.74
C14:1	0.81	0.66	0.045	0.08
C15:0	0.80	0.72	0.038	0.30
*cis*-10 C15:1	0.05	0.04	0.005	0.13
C16:0	17.77	17.45	0.129	0.22
*cis*-9 C16:1	3.21	3.39	0.112	0.42
C17:0	0.91	0.89	0.013	0.36
C17:1	0.57	0.55	0.013	0.44
C18:0	13.15	9.87	1.342	0.22
*cis*-9 C18:1	34.81	36.15	0.676	0.33
*trans*-9 C18:1	5.73	7.69	1.604	0.55
*trans*-9,12 C18:2	0.15	0.08	0.021	0.15
*cis*-9,12 C18:2	2.83	3.58	0.239	0.14
C20:0	0.07	0.04	0.008	0.08
*cis*-6,9,12 C18:3	0.05	0.03	0.006	0.10
*cis*-11 C20:1	0.38	0.38	0.014	0.85
*cis*-9,12,15 C18:3	0.31	0.27	0.022	0.47
C21:0	0.04	0.03	0.004	0.06
*cis*-11,14 C20:2	0.04	0.02	0.004	0.02
C22:0	0.04	0.02	0.005	0.05
*cis*-8,11,14 C20:3	0.09	0.06	0.012	0.26
*cis*-13 C22:1	0.04	0.02	0.004	0.11
*cis*-11,14,17 C20:3	0.21	0.16	0.017	0.23
*cis*-5,8,11,14 C20:4	0.08	0.09	0.015	0.58
C23:0	0.04	0.02	0.004	0.10
*cis*-13,16 C22:2	0.02	0.01	0.002	0.33
C24:0	0.05	0.04	0.004	0.10
*cis*-5,8,11,14,17 C20:5	0.05	0.03	0.004	0.04
*cis*-15 C24:1	0.06	0.03	0.006	0.04
*cis*-4,7,10,13,16,19 C22:6	0.07	0.04	0.008	0.06
SFAs	50.45	46.70	1.504	0.22
UFAs	49.55	53.30	1.504	0.22
MUFAs	45.67	48.91	1.446	0.27
PUFAs	3.88	4.39	0.202	0.24
De novo	19.22	19.02	0.367	0.69
Mixed	20.98	20.84	0.137	0.63
Preformed	59.80	60.14	0.407	0.79
C ≤ 16	37.00	36.47	0.415	0.54
C > 16	63.00	63.53	0.415	0.54

^1^*SFAs* Saturated fatty acids, *UFAs* Unsaturated fatty acids, *MUFAs* Monounsaturated fatty acids, *PUFAs* Polyunsaturated fatty acids. De novo FAs (< 16 C) originate from de novo synthesized in the mammary gland, preformed FAs (> 16 C) originate from plasma, and mixed FAs (16 C) originate from both sources. *SEM* Standard error of means. CON (*n* = 15) and BAS (*n* = 17), without and with supplementing 20 g/d of bile acids, respectively

**Table 5:** Effects of supplementing bile acids on milk fatty acid yields at d 21 postpartum in transition dairy cows

Items, g/d^1^	Treatments		SEM	*P*-value
	CON	BAS		
C4:0	32.83	40.72	1.783	0.03
C6:0	20.55	25.54	1.141	0.03
C8:0	12.94	18.27	0.959	< 0.01
C10:0	32.98	40.92	2.986	0.19
C11:0	1.25	1.31	0.096	0.77
C12:0	40.59	54.98	2.588	< 0.01
C13:0	1.87	2.17	0.131	0.27
C14:0	193.24	239.01	8.095	< 0.01
C14:1	15.37	15.85	1.082	0.83
C15:0	15.45	17.90	1.069	0.26
*cis*-10 C15:1	0.99	0.92	0.078	0.68
C16:0	342.13	422.23	15.420	< 0.01
*cis*-9 C16:1	62.37	82.39	3.918	< 0.01
C17:0	17.48	21.48	0.722	< 0.01
C17:1	11.05	13.40	0.559	0.03
C18:0	251.05	239.41	31.152	0.86
*cis*-9 C18:1	673.96	873.91	35.144	< 0.01
*trans*-9 C18:1	108.43	186.08	34.654	0.27
*trans*-9,12 C18:2	2.68	1.90	0.399	0.34
*cis*-9,12 C18:2	56.42	88.39	6.575	0.01
C20:0	1.35	1.10	0.161	0.45
*cis*-6,9,12 C18:3	0.90	0.66	0.105	0.30
*cis*-11 C20:1	7.44	9.17	0.468	0.07
*cis*-9,12,15 C18:3	5.87	6.61	0.543	0.51
C21:0	0.82	0.65	0.061	0.22
*cis*-11,14 C20:2	0.82	0.59	0.081	0.18
C22:0	0.81	0.58	0.076	0.18
*cis*-8,11,14 C20:3	1.63	1.54	0.238	0.86
*cis*-13 C22:1	0.67	0.55	0.060	0.37
*cis*-11,14,17 C20:3	3.82	3.86	0.352	0.96
*cis*-5,8,11,14 C20:4	1.54	2.30	0.366	0.31
C23:0	0.70	0.54	0.070	0.26
*cis*-13,16 C22:2	0.31	0.33	0.034	0.69
C24:0	1.01	0.90	0.068	0.42
*cis*-5,8,11,14,17 C20:5	0.83	0.66	0.076	0.28
*cis*-15 C24:1	1.08	0.74	0.101	0.10
*cis*-4,7,10,13,16,19 C22:6	1.32	0.87	0.140	0.11
SFAs	967.06	1,127.71	49.337	0.11
UFAs	957.49	1,290.74	60.334	< 0.01
MUFAs	881.36	1,183.02	55.552	< 0.01
PUFAs	76.13	107.72	6.510	0.01
De novo	368.07	457.60	16.720	< 0.01
Mixed	404.50	504.62	18.846	< 0.01
Preformed	1,151.99	1,456.23	53.840	< 0.01
C ≤ 16	710.20	879.83	31.080	< 0.01
C > 16	1,214.36	1538.62	57.190	< 0.01

^1^*SFAs* Saturated fatty acids, *UFAs* Unsaturated fatty acids, *MUFAs* Monounsaturated fatty acids, *PUFAs* Polyunsaturated fatty acids. De novo FAs (< 16 C) originate from de novo synthesized in the mammary gland, preformed FAs (> 16 C) originate from plasma, and mixed FAs (16 C) originate from both sources. *SEM* Standard error of means. CON (*n* = 15) and BAS (*n* = 17), without and with supplementing 20 g/d of bile acids, respectively

### Effects of BA supplementation on fecal bacterial community and function in postpartum dairy cows

**Fig. 1:**
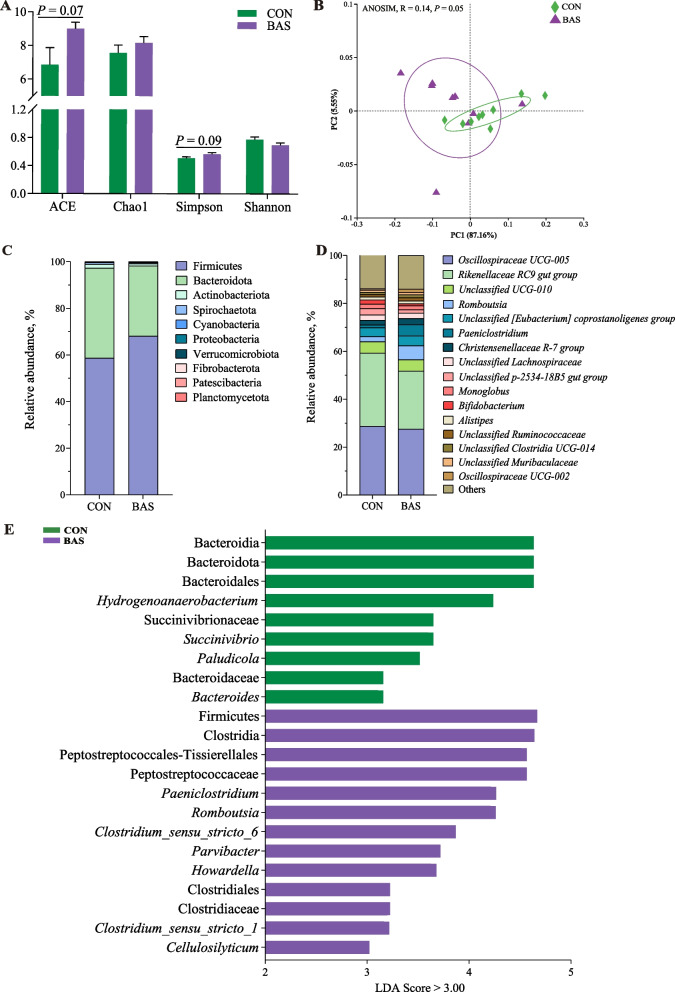
Fecal microbial community composition and diversity between the CON and BAS groups (*n* = 9). **A** The ACE, Chao1, Simpson, and Shannon indices of fecal microbiota were analyzed using the Wilcoxon rank sum test for differences. **B** Principal coordinates analysis (PCoA) used Similarity Analysis (ANOSIM) at the phylum level to determine the statistical significance of 999 permutations. **C** The relative abundance of bacterial phyla in the feces between the CON and BAS groups. **D** The relative abundance of bacterial genera in the feces between the CON and BAS groups. **E** Linear discriminant analysis effect size (LEfSe) between the CON and BAS groups. CON and BAS, without and with supplementing 20 g/d of bile acids, respectively

**Fig. 2:**
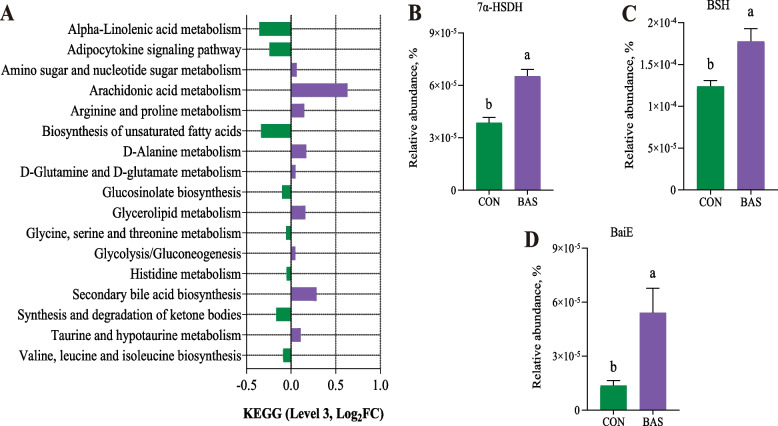
Functional prediction of fecal microbial KEGG pathway and bile acid conversion related enzymes between the CON and BAS groups (*n* = 9). **A** KEGG pathways (Level 3) of fecal microbiota between the CON and BAS groups. Green represents an increase in the CON group, while purple represents an increase in the BAS group. **B** 7α-hydroxysteroid dehydrogenase (7α-HSDH) relative abundance percentage between the CON and BAS groups. **C** Bile salt hydrolase (BSH) relative abundance percentage between the CON and BAS groups. **D** Bile acid inducible E (BaiE) relative abundance percentage between the CON and BAS groups. CON and BAS, without and with supplementing 20 g/d of bile acids, respectively. ^a^^,^^b^Different superscripts differ significantly (*P* < 0.05)

The BAS group tended to have greater ACE (*P* = 0.07) and Simpson values (*P* = 0.09) than the CON group (Fig. 1A). The PCoA and ANOSIM (*R* = 0.14, *P* = 0.05) showed a trended significant difference in the beta diversity between these two groups (Fig. 1B). A total of 10 phyla and 173 genera were detected in the feces of the two groups. Firmicutes and Bacteroidota were the two most abundant phyla, accounting for 63.40% ± 0.10% and 34.30% ± 0.01% of the total sequences, respectively (Fig. 1C). *Oscillospiraceae UCG-005* and *Rikenellaceae-RC9 gut group* were the two most abundant genera, accounting for 28.81% ± 0.10% and 27.40% ± 0.07% of the total sequences, respectively (Fig. 1D). LEfSe analysis identified that 13 taxa were greater and 9 were lower in the BAS group than in the CON group (Fig. 1E). Among that, the relative abundance of *Romboutsia*, *Clostridium sensu_stricto_6*, and *Clostridium sensu_stricto_1* was greater in the BAS group at the genus level. The PICRUSt2 functional prediction indicated that at the level 3 KEGG pathway, 9 pathways were up-regulated in the BAS group compared with the CON group, especially SBA biosynthesis (*P* = 0.03) and taurine and hypotaurine metabolism (*P* = 0.05), and 8 pathways were down-regulated in the BAS group (Fig. 2A). In addition, the relative abundance of bacterial 7α-HSDH (*P* < 0.01), BSH (*P* < 0.01), and BaiE (*P* = 0.02) enzymes was also greater in the BAS group than in the CON group (Fig. 2B–D).

### Effects of BA supplementation on plasma and fecal BA profiles in postpartum dairy cows

**Fig. 3:**
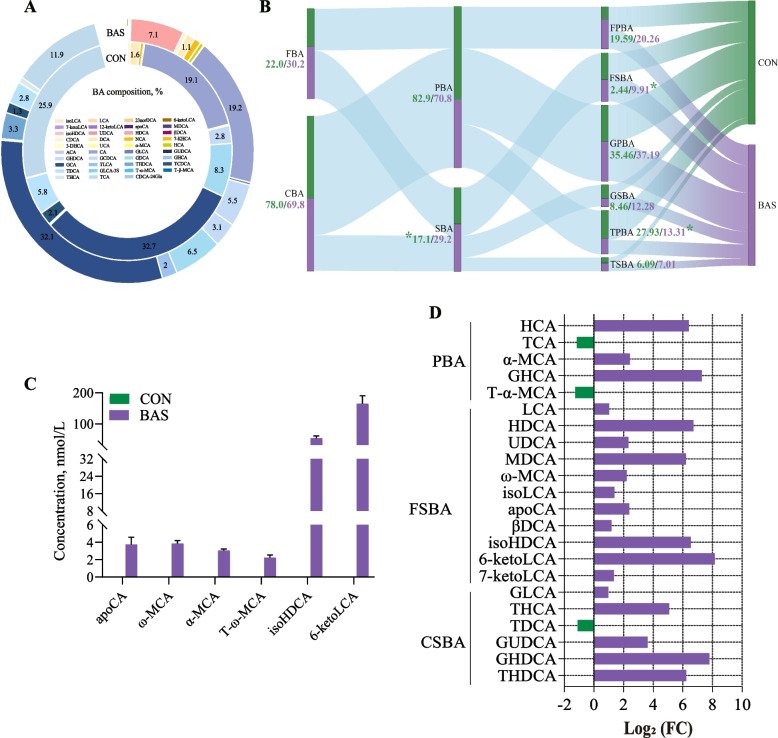
Plasma bile acid profiles and flows between the CON and BAS groups (*n* = 9). **A** The relative abundance percentage of plasma bile acids between the CON and BAS groups. **B** The flow between different kinds of bile acids in the plasma of the CON and BAS groups. The numbers represent the proportions of each type of bile acids, and “*” indicates significant differences (*P* < 0.05). *FBA* Free bile acid, *FPBA* Free primary bile acid, *GPBA* Glycine primary bile acid, *GSBA* Glycine secondary bile acid, *TSBA* Taurine secondary bile acid. **C** Bile acids detected exclusively in the BAS group compared to CON group. **D** The relative abundance of bile acids in the plasma between the CON and BAS groups is significantly different. Green represents an increase in the CON group, while purple represents an increase in the BAS group. *HCA* Hyocholic acid, *TCA* Taurocholic acid, *T-α-MCA* Tauro α-muricholic acid, *LCA* Lithocholic acid, *UDCA* Ursodeoxycholic acid, *isoLCA* Isoallolithocholic acid, *βDCA* 3-Epideoxycholic acid, *7-ketoLCA* 7-Ketolithocholic acid, *GLCA* Glycolithocholic acid, *TDC*A Taurodeoxycholic acid. CON and BAS, without and with supplementing 20 g/d of bile acids, respectively

In total, 39 kinds of BAs were commonly detected in the plasma of both groups (Fig. 3A). Glycocholic acid, cholic acid, and taurocholic acid were the main BAs in these two groups, accounting for 32.37% ± 0.01%, 19.18% ± 0.01%, and 18.97% ± 0.01% of the total BA, respectively. In terms of the BA categories, the BAS group had greater SBA (*P* = 0.02) and free secondary BA (FSBA) (*P* < 0.01) proportions but a lower taurine primary bile acid (TPBA) proportion (*P* = 0.05) than the CON group (Fig. 3B). No significant differences were found in the proportions of other BA categories. However, six unique BAs, including apocholic acid, ω-muricholic acid, α-muricholic acid, tauro α-Muricholic acid, isohyodeoxycholic acid (isoHDCA), and 6-ketolithic acid (6-ketoLCA), were only found in the BAS group (Fig. 3C). Analysis of differences in plasma BA composition showed that 19 kinds of BAs were greater (*P* < 0.01), and 3 kinds of BAs were lower (*P* < 0.01) in the BAS group than in the CON group (Fig. 3D). Among that, 11 kinds of FSBA and 6 kinds of conjugated secondary BA (CSBA), including hyodeoxycholic acid (HDCA) (*P* < 0.01), murideoxycholic acid (MDCA) (*P* < 0.01), isoHDCA (*P* < 0.01), 6-ketoLCA (*P* < 0.01), and taurohyocholic acid (THCA) (*P* < 0.01), were greater in the BAS group than in the CON group.

**Fig. 4:**
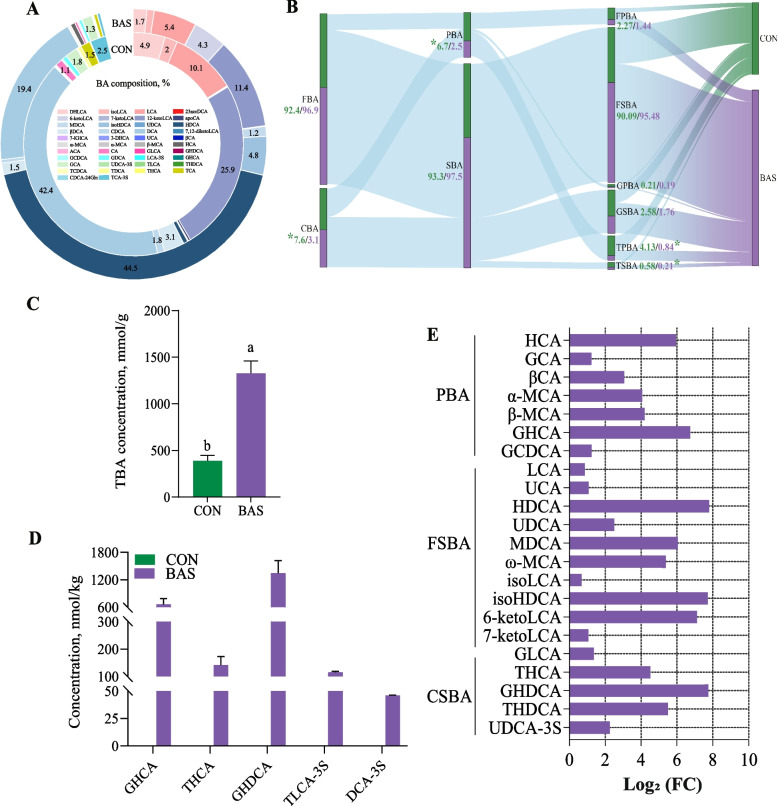
Fecal bile acid profiles and flows between the CON and BAS groups (*n* = 9). **A** The relative abundance percentage of fecal bile acids between the CON and BAS groups. **B** The flow between different kinds of bile acids in the feces. The numbers represent the proportion of each type of bile acid, and “*” indicates significant differences (*P* < 0.05). *FBA* Free bile acid, *FPBA* Free primary bile acid, *GPBA* Glycine primary bile acid, *GSBA* Glycine secondary bile acid, *TSBA* Taurine secondary bile acid. **C** The total bile acid concentration in the feces of the CON and BAS groups. **D** Bile acids detected exclusively in the BAS group compared to CON group. *TLCA-3S* Taurolithocholic acid-3-sulfate, *DCA-3S* Deoxycholic acid-3-sulfate. **E** The relative abundance of BA in the feces between CON and BAS groups is significantly different. Purple represents an increase in the BAS group. *HCA* Hyocholic acid, *GCA* Glycocholic acid, *βCA* 3β-Cholic acid, *α-MCA* α-Muricholic acid, *β-MCA* β-Muricholic acid, *GCDCA* Glycochenodeoxycholic acid, *LCA* Lithocholic acid, *UCA* Ursocholic acid, *UDCA* Ursodeoxycholic acid, *ω-MCA* ω-Muricholic acid, *isoLCA* Isoallolithocholic acid, *7-ketoLCA* 7-Ketolithocholic acid, *GLCA* Glycolithocholic acid, *UDCA-3S* Ursodeoxycholic acid 3-sulfate. CON and BAS, without and with supplementing 20 g/d of bile acids, respectively. ^a,b^Different superscripts differ significantly (*P* < 0.05)

In total, 42 kinds of BAs were commonly detected in the feces of both groups (Fig. 4A). Taurocholic acid, dehydrocholic acid, and HDCA were the main BAs in these two groups, accounting for 34.50% ± 0.02%, 24.60% ± 0.01%, and 14.70% ± 0.004% of the total BA. In terms of the BA categories, the BAS group had lower conjugated bile acid (CBA) (*P* = 0.01), PBA (*P* = 0.04), TPBA (*P* = 0.02), and taurine secondary bile acid (*P* = 0.01) proportions than in the CON group (Fig. 4B). In the fecal BA pool, the total BA concentration was greater in the BAS group than in the CON group (Fig. 4C, *P* < 0.01). However, five unique BAs, including GHCA, THCA, glycohyodeoxycholic acid (GHDCA), taurolithocholic acid-3-sulfate, and deoxycholic acid-3-sulfate, were only found in the BAS group (Fig. 4D). Analysis of differences in fecal BA composition showed that 22 kinds of BAs were greater (*P* < 0.01) in the BAS group than in the CON group (Fig. 4E). Among that, 10 kinds of FSBA and 5 kinds of CSBA, including HDCA (*P* < 0.01), MDCA (*P* < 0.01), isoHDCA (*P* < 0.01), 6-ketoLCA (*P* < 0.01), THCA (*P* < 0.01), were greater in the BAS group than in the CON group .

**Fig. 5:**
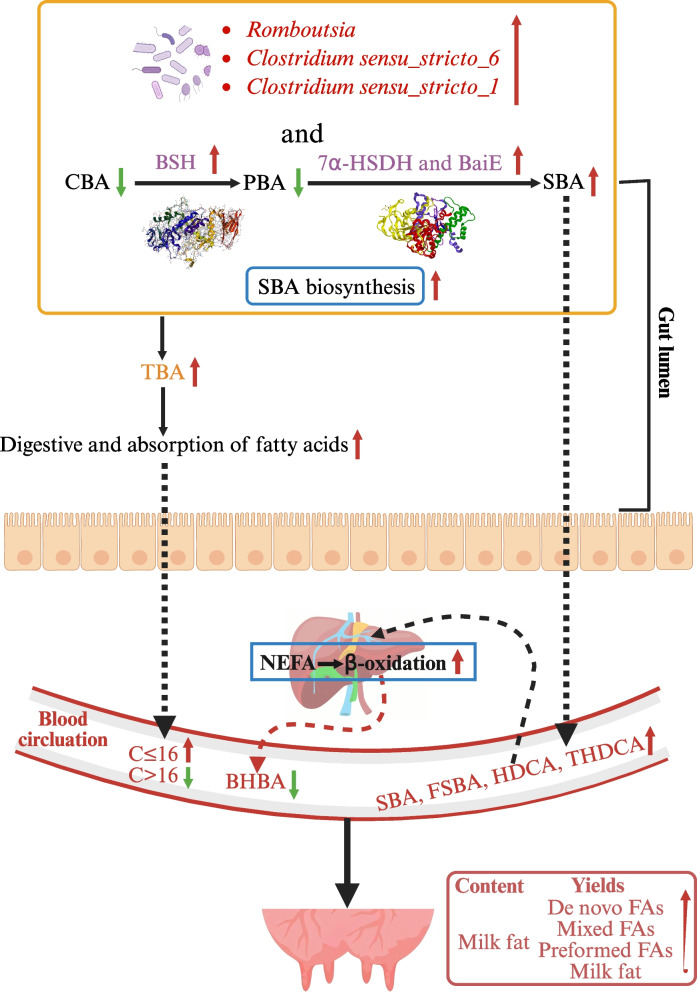
Process of effects of supplementing bile acids on the production performance, fatty acid and bile acid composition, and gut microbiota in transition dairy cows. 1. After BA supplementation, the digestion and absorption of FAs to the intestine leads to increase of medium/short-chain FAs (C ≤ 16 FAs) in the plasma. The taurine- and glycine-conjugated BA in the plasma of might up-regulate the de novo synthesized of FAs, resulting in an increase in de novo synthesized of medium/short-chain FA in milk, the increased of TBA in the gut may increase the absorption rate of long-chain FAs, which ultimately contributes to the increased milk fat content and yields, and de novo FAs, mixed FAs, preformed FAs yields. 2. After BA supplementation, the abundance of microorganisms beneficial for BA conversion, such as *Romboutsia*, *Clostridium sensu_stricto_6*, and *Clostridium sensu_stricto_1*, and the increase in bile salt hydrolase (BSH) abundance, leads to a reduction in the proportion of conjugated bile acid (CBA) in the intestine. The increase in abundance of 7α-hydroxysteroid dehydrogenase (7α-HSDH) and bile acid inducible E (BaiE) leads to a decrease in the proportion of primary bile acid (PBA) in the intestine, and an increase in the proportion of secondary bile acid (SBA) in the plasma. 3. After BA supplementation, SBA, free secondary bile acid (FSBA), and some independent BAs such as hyodeoxycholic acid (HDCA) and taurohyodeoxycholic acid (THDCA) entering the liver can enhance the complete oxidation ability of nonesterified fatty acid (NEFA), resulting in decreased BHBA concentration. Biorender software was used to create this illustration, red represents rise, green represents fall

## Discussion

Recent studies had demonstrated that BAs can regulate glucose and lipid metabolism in humans and rodents [16–18]. In monogastric and aquatic animals, BAs had also been used as feed additives to improve hepatic lipid metabolism and performance [47, 48]. However, few studies investigated the effects of BAs in ruminants [31, 32], especially in transition dairy cows. To our knowledge, this is the first study supplementing BAs in transition dairy cows to investigate the effects on production performance and body metabolism.

BAs, which originate from cholesterol in the liver, are stored in the gallbladder and then secreted into the intestine by the stimulating of cholecystokinin. As an amphiphilic molecule composed of hydrophilic and hydrophobic groups, BAs are natural emulsifiers that effectively emulsify lipids. This increases the contact area between lipase and lipids and form mixed micelles with phospholipids, thereby promoting the dissolution of monoglycerides and FAs as well as the digestion and absorption of dietary lipids in the small intestine [49–51]. This might be one of the main reasons for the increased proportions of plasma medium/short-chain FAs such as C12:0, C14:0, and C16:0 in the BAS group of our study.

In the mammary gland, the acetate and BHBA were used to de novo synthesize medium/short-chain FAs with 4–16 carbon atoms [52]. In this study, the ruminal acetate concentration were similar between these two groups. Previous studies have shown that taurine- and glycine-conjugated BA might up-regulate the expression of key transcription factors and rate-limiting enzymes for the de novo synthesized of FAs by activating the sphingosine-1-phosphate receptor 2 (*S1PR2*) gene [53–55]. Similarly, the plasma proportions of GHDCA, taurohyodeoxycholic acid (THDCA), THCA, and glycoursodeoxycholic acid (GUDCA) were significantly increased in the BAS group, which can be one of the main reasons for increased de novo synthesized medium/short-chain FAs yields such as C8:0, C12:0, C14:0, and C16:0 in the BAS group of our study. At the same time, the increased TBA in the gut of the BAS group might increase the absorption rate of long-chain FAs [56], which ultimately contributed to the increased yields of preformed FAs in milk and milk fat content and yields. Similarly, previous studies also reported that BA supplementation improved production performance in mid-lactation cows and dairy goats [31, 32]. In addition, the simultaneous increase in de novo synthesized medium/short-chain FAs, mixed FAs, and preformed FAs yields could lead to the similar proportions of these FAs in milk.

The gut microbiota contributed uniquely to the diversity of BA profiles mainly through the enzymes, including BSH, 7α-HSDH, and BaiE (responsible for encoding enzymes involved in the initial steps of the BA 7α-dehydroxylation pathway) [57]. The BAS group had a greater relative abundance of *Romboutsia*, which was reported to have the potential enzyme function of expressing BSH and the ability to accelerate SBA synthesis [58]. Similarly, the increased relative abundance of *Clostridium* such as *Clostridium sensu_stricto-6* and *Clostridium sensu_stricto-1* in the BAS group are also commonly known to have the ability of BA transformation with 7α-HSDH enzymes [59, 60]. In this study, microbial functional prediction analysis showed an increased relative abundance of BSH, 7α-HSDH, and BaiE enzymes in the BAS group, indicating a wide conversion of PBA to SBA in the intestine through stronger microbial hydrolysis and dehydrogenation. This process might be one of the main reasons for increased reabsorption of SBA in the plasma and decreased PBA in the feces. The reduction of PBA in the enterohepatic circulation system might be beneficial for the clearance of liver cholesterol and improving lipid metabolism [61]. Meanwhile, microbial functional prediction analysis also reported up-regulated SBA synthesis and taurine and hypotaurine metabolism in the BAS group, further supporting the inference that gut microbiota, such as *Clostridium* and *Romboutsia*, were widely involved in BA conversion and metabolism in the gastrointestinal tract. In accordance, one of our previous studies also found enhanced pathways related to lipid and BA metabolism and enzymes related to BA transformation of gut microbiota in dairy goats supplemented with BAs [31].

The liver has an orchestrated mechanism for regulating the hepatocellular mechanism by BAs. One of the known pathways is that the activation of hepatic farnesol X receptor by BAs, especially SBA, can further activate peroxisome proliferators-activated receptor-related pathways and then enhance the oxidation of FAs in the liver [62–64]. In this study, the increased SBA, especially HDCA, isoHDCA, GHDCA, and THDCA in plasma and feces, might represent increased SBA reabsorbing and transferring to the liver. This process could promote the completely β-oxidation of NEFA resulting in lower plasma BHBA concentration in the cows supplemented with BAs. Similarly, previous studies also reported that BA supplementation can reduce hepatic lipid deposition by altering enzymes involved in BA conversion of gut microbiota and enhancing β-oxidation of lipid in the liver [47, 48]. However, further studies with liver samples or in the primary bovine hepatic cells are needed to confirm this inference.

The synthesis and excretion of BAs are the main metabolic pathways for cholesterol and lipids [65]. In order to maintain the homeostasis of the BA pool, the amount of BAs synthesized by the liver should be equal to the amount of BAs excreted in feces [66]. Although the fecal TBA concentration was greater in cows supplemented with BAs, the plasma TBA concentration was similar in these two groups, suggesting that the body has the ability to maintain the homeostasis of the amount of TBA in the systemic circulation, but the BA profiles fluctuate depending on the metabolic requirements of the whole body. A recent study also reported increased excretion of BAs in feces after BA supplementation, thereby reducing the lipid toxicity of plasma cells [48].

BSH can modify PBA through 7α/β-dehydroxylation with proteins of the *bai* operon, and then isomerized by the family of hydroxysteroid dehydrogenases to produce SBA [67]. This study also found that the changed SBA profiles in the plasma were mainly coming from the PBA in the intestine undergoing one-step catalysis by BSH, and the changed SBA in feces were mainly derived the PBA in the intestine undergoing two-step catalysis by BSH and 7α-HSDH or BaiE. In addition, Furthermore, most of the unique BA profiles in plasma were precursors of the unique BA profiles in the feces. This information was consistent with the known physiological transformation of BAs in the intestine and enterohepatic circulation [68].

Although our investigation attempted to comprehensively understand the potential contribution of microbe and BAs interaction to the production performance and body metabolism in transition dairy cows with BA supplementation, we also recognized that our research had some limitations. Firstly, this study was conducted on a commercial farm and could not obtain individual daily dry matter intake and reliable total-tract apparent digestibility data, and we speculated that the changed production performance was due to the digestion and absorption in the gastrointestinal tract by BA supplementation. Secondly, the plasma BA profiles were used to represent the body's BA metabolism, and fecal BA profiles and microbiota were used to represent intestinal BAs and microbiota, which are both critical limitations. In addition, the direct evidence of BAs affecting hepatic lipid β-oxidation needed further investigations. Overall, our limitation ultimately lied in the inability to obtain samples of dairy cow intestinal, mammary gland, and liver tissue samples. More work is needed to study the BA's transcriptional process and role in whole-body metabolism with BA supplementation. This will help us to understand how to develop new nutritional regulation strategies for transition dairy cows.

## Conclusions

In summary, BA supplementation in transition dairy cows significantly increased postpartum milk FAs yields, milk fat content and yields, which might due to enhanced de novo synthesized and absorbed FAs in the mammary gland. BA supplementation also increased proportions of plasma SBA and reduced the BHBA concentration, which might be attributed to increased activity of enzymes related to SBA synthesized of gut microbiota and then promoted complete oxidation of NEFA in the liver (Fig. 5). Meanwhile, dairy cows might have the ability to maintain BAs homeostasis at the systemic level as the plasma TBA concentrations was stable but the fecal TBA excretion was enhancive with BA supplementation despite the BA profiles were changed. This study provides a new theoretical and technical support for applying BAs in transition dairy cows, although more research are needed to investigate the molecular mechanisms with more tissue samples.

## Supplementary Information

Additional file 1: Table S1 Feed ingredients and nutrient composition of prepartum, postpartum, and lactating diets. Table S2 Effects of supplementing bile acids on rumen fermentation at d 14 postpartum in transition dairy cows. Table S3 Effects of supplementing bile acids on plasma bile acid individual and categories on d 21 postpartum in transition dairy cows. Table S4 Effects of supplementing bile acids on fecal bile acid individual and categories on d 21 postpartum in transition dairy cows.

Additional file 2: Fig. S1 Rumen microbial community composition and diversity between CON and BAS groups (n = 9). A The ACE, Chao1, Simpson, and Shannon indices of rumen microbiota were analyzed using the Wilcon rank sum test for differences. B Principal coordinates analysis (PCoA) used Similarity Analysis (ANOSIM) at the phylum level to determine the statistical significance of 999 permutations. C The relative abundance of bacterial phylum in the rumen of the CON and BAS groups. D The relative abundance of bacterial genera in the rumen of the CON and BAS groups. CON and BAS, without and with supplementing 20 g/d of bile acids, respectively.

## Data Availability

Raw sequencing data of all 16S rRNA sequences have been deposited into the NCBI Sequence Read Archive (SRA) under accession numbers PRJNA1155809 and PRJNA1150763, respectively.

## Abbreviations

apoCA: Apocholic acid
BAs: Bile acids
CBA: Conjugated bile acid
DCA-3S: Deoxycholic acid-3-sulfate
FAs: Fatty acids
FBA: Free bile acid
FPBA: Free primary bile acid
FSBA: Free secondary bile acid
GCA: Glycocholic acid
GCDCA: Glycochenodeoxycholic acid
GHCA: Glycohyocholic acid
GHDCA: Glycohyodeoxycholic acid
GLCA: Glycolithocholic acid
GPBA: Glycine primary bile acid
GSBA: Glycine secondary bile acid
GUDCA: Glycoursodeoxycholic acid
HCA: Hyocholic acid
HDCA: Hyodeoxycholic acid
isoHDCA: Isohyodeoxycholic acid
isoLCA: Isoallolithocholic acid
LCA: Lithocholic acid
MDCA: Murideoxycholic acid
PBA: Primary bile acid
SBA: Secondary bile acid
TCA: Taurocholic acid
TDCA: Taurodeoxycholic acid
THCA: Taurohyocholic acid
THDCA: Taurohyodeoxycholic acid
TLCA-3S: Taurolithocholic acid-3-sulfate
TPBA: Taurine primary bile acid
TSBA: Taurine secondary bile acid
T-α-MCA: Tauro α-muricholic acid
T-ω-MCA: Tauro ω-muricholic acid
UCA: Ursocholic acid
UDCA: Ursodeoxycholic acid
UDCA-3S: Ursodeoxycholic acid 3-sulfate
6-ketoLCA: 6-Ketolithocholic acid
7-ketoLCA: 7-Ketolithocholic acid
α-MCA: α-Muricholic acid
βCA: 3β-Cholic acid
βDCA: 3-Epideoxycholic acid
β-MCA: β-Muricholic acid
ω-MCA: ω-Muricholic acid
