# Supplementary material for: Effects of supplementing bile acids on the production performance, fatty acid and bile acid composition, and gut microbiota in transition dairy cows
Source: J Anim Sci Biotechnol. 2025 Jun 12;16:83. doi: 10.1186/s40104-025-01207-8 (PMC12160099; doi:10.1186/s40104-025-01207-8)
Supplement: Supplementary file 1 — Additional file 1: Table S1 Feed ingredients and nutrient composition of prepartum, postpartum, and lactating diets. Table S2 Effects of supplementing bile acids on rumen fermentation at d 14 postpartum in transition dairy cows. Table S3 Effects of supplementing bile acids on plasma bile acid individual and categories on d 21 postpartum in transition dairy cows. Table S4 Effects of supplementing bile acids on fecal bile acid individual and categories on d 21 postpartum in transition dairy cows. [file 40104_2025_1207_MOESM1_ESM.docx]

**Table S1** Feed ingredients and nutrient composition of prepartum, postpartum, and lactating diets

| **Ingredients** | **% of DM^1^** | | |
| --- | --- | --- | --- |
|  | **Prepartum** | **Postpartum** | **Lactating** |
| Oat hay | 3.30 | 1.00 | 1.00 |
| Alfalfa hay | 1.00 | 2.80 | 2.80 |
| Corn silage | 13.00 | 15.00 | 15.00 |
| Canola meal | 1.00 | 0.70 | 0.70 |
| Beet granules | 1.00 | 0.70 | 0.70 |
| Whole cottonseed | - | 1.30 | 1.30 |
| Steam-flaked corn | 2.10 | 2.00 | 2.00 |
| Crushed corn | 0.00 | 2.50 | 2.50 |
| Soybean meal | 1.80 | 2.60 | 2.60 |
| Extruded soybean | - | 0.60 | 0.60 |
| Premix^2^ | 0.70 | 0.70 | 0.70 |
| Rumen-protected glucose | 0.10 | 0.10 | 0.10 |
| Rumen-protected methionine | 0.03 | 0.03 | 0.03 |
| Chlorin soybean meal | 0.25 | 0.00 | 0.00 |
| Choline chloride | 0.03 | 0.025 | 0.025 |
| Salt | - | 0.20 | 0.20 |
| DM | 48.57 ± 0.89 | 50.01 ± 0.85 | 48.51 ± 0.63 |
| Chemical analysis, % of DM ± SD^3^ | | |  |
| Starch | 19.04 ± 1.21 | 21.83 ± 0.49 | 25.28 ± 0.36 |
| CP | 15.40 ± 0.13 | 18.14 ± 0.25 | 18.82 ± 0.33 |
| EE | 3.19 ± 0.12 | 4.27 ± 0.07 | 4.56 ± 0.39 |
| NDF | 39.77 ± 1.03 | 37.44 ± 2.15 | 40.52 ± 1.76 |
| ADF | 23.68 ± 0.94 | 15.57 ± 0.67 | 14.13 ± 0.29 |
| NFC^4^ | 32.36 ± 0.36 | 30.85 ± 0.63 | 28.41 ± 0.65 |
| Ash | 9.28 ± 0.14 | 9.30 ± 0.05 | 7.69 ± 0.10 |
| Ca | 0.59 ± 0.002 | 0.48 ± 0.01 | 0.44 ± 0.01 |
| P | 0.35 ± 0.02 | 0.39 ± 0.002 | 0.44 ± 0.01 |

^1^ Prepartum was fed from d 21 ± 3 before the expected calving date to calving; Postpartum was fed from calving d 0 to 21; Lactating was fed from d 22 to d 60

^2^ Premix contained (per kg of diet): 250,000 IU of vitamin A, 49,000 IU of vitamin D_3_, 2,600 IU of vitamin E, 100 mg of CU, 500 mg of Zn, 300 mg of Mn, 11 mg of I, 5mg of Se, and 9 mg of Co

^3^ *CP* Crude protein, *EE* Ether extract, *NDF* Neutral detergent fiber, *ADF* Acid detergent fiber, *Ca* Calcium, *P* Phosphorus. *SD* Standard deviation

^4^ NFC = 100% − (% NDF + % CP + % EE + % Ash)

**Table S2** Effects of supplementing bile acids on rumen fermentation at d 14 postpartum in transition dairy cows

| **Items^1^** | **Treatments** | | **SEM** | ***P*-value** |
| --- | --- | --- | --- | --- |
|  | **CON** | **BAS** |  |  |
| Rumen pH | 6.71 | 6.73 | 0.051 | 0.88 |
| Ammonia N, mg/dL | 14.67 | 15.42 | 0.904 | 0.89 |
| Total VFAs, mmol/L | 105.60 | 113.80 | 3.215 | 0.21 |
| Acetate, mmol/L | 63.54 | 70.53 | 1.983 | 0.08 |
| Propionate, mmol/L | 26.42 | 26.00 | 0.975 | 0.84 |
| Isobutyrate, mmol/L | 0.81 | 0.91 | 0.043 | 0.26 |
| Butyrate, mmol/L | 12.03 | 13.25 | 0.546 | 0.27 |
| Isovalerate, mmol/L | 1.48 | 1.65 | 0.088 | 0.34 |
| Valerate, mmol/L | 1.33 | 1.46 | 0.055 | 0.25 |

^1^ *TVFAs* Total volatile fatty acids. *SEM*, Standard error of means. CON (*n* = 15) and BAS (*n* = 17), without and with supplementing 20 g/d of bile acids, respectively

**Table S3** Effects of supplementing bile acids on plasma bile acid individual and categories on d 21 postpartum in transition dairy cows

| **Items^1^** | **Treatments** | | **SEM** | ***P*-value** |
| --- | --- | --- | --- | --- |
|  | **CON** | **BAS** |  |  |
| BA individual, nmol/L | | | | |
| HCA | 3.52 | 297.66 | 43.862 | < 0.01 |
| TCA | 16594.76 | 7498.30 | 2289.064 | 0.04 |
| α-MCA | 0.51 | 2.76 | 0.316 | < 0.01 |
| GHCA | 8.17 | 1267.26 | 240.435 | 0.01 |
| T-α-MCA | 19.34 | 8.12 | 2.401 | 0.01 |
| LCA | 6.51 | 13.25 | 1.391 | 0.01 |
| HDCA | 42.62 | 4468.24 | 663.130 | < 0.01 |
| UDCA | 1.51 | 7.64 | 1.000 | < 0.01 |
| MDCA | 1.47 | 108.6 | 18.306 | < 0.01 |
| ω-MCA | 0.51 | 2.37 | 0.373 | 0.02 |
| isoLCA | 0.84 | 2.18 | 0.271 | 0.01 |
| apoCA | 0.51 | 2.67 | 0.452 | 0.02 |
| βDCA | 4.49 | 10.12 | 1.340 | 0.04 |
| isoHDCA | 0.51 | 47.74 | 7.252 | < 0.01 |
| 6-ketoLCA | 0.51 | 146.39 | 22.628 | < 0.01 |
| 7-ketoLCA | 9.66 | 24.60 | 2.815 | 0.01 |
| GLCA | 108.16 | 211.17 | 24.702 | 0.04 |
| THCA | 12.68 | 430.90 | 77.224 | 0.01 |
| TDCA | 3735.35 | 1753.44 | 470.334 | 0.03 |
| GUDCA | 0.85 | 10.48 | 1.602 | < 0.01 |
| GHDCA | 15.47 | 3433.25 | 532.840 | < 0.01 |
| THDCA | 27.93 | 2097.35 | 320.679 | < 0.01 |
| BA categories % |  |  |  |  |
| FBA | 22.03 | 30.17 | 0.351 | 0.21 |
| CBA | 77.97 | 69.83 | 1.078 | 0.69 |
| PBA | 82.90 | 70.76 | 0.915 | 0.48 |
| SBA | 17.10 | 29.24 | 0.305 | 0.02 |
| FPBA | 19.59 | 20.26 | 0.267 | 0.90 |
| FSBA | 2.44 | 9.91 | 0.135 | < 0.01 |
| GPBA | 35.46 | 37.19 | 0.559 | 0.87 |
| GSBA | 8.46 | 12.28 | 0.135 | 0.12 |
| TPBA | 27.93 | 13.31 | 0.416 | 0.05 |
| TSBA | 6.09 | 7.01 | 0.092 | 0.60 |

^1^ *BAs* Bile acids, *HCA* Hyocholic acid, *TCA* Taurocholic acid, *α-MCA* α-Muricholic acid, *GHCA* Glycohyocholic acid, *T-α-MCA* Tauro-α-muricholic acid, *LCA* Lithocholic acid, *HDCA* Hyodeoxycholic acid, *UDCA* Ursodeoxycholic acid, *MDCA* Murideoxycholic acid, *ω-MCA* ω-Muricholic acid, *isoLCA* isoallolithocholic acid, *apoCA* apocholic acid, *βDCA* 3-Epideoxycholic acid, *isoHDCA* isohyodeoxycholic acid, *6-ketoLCA* 6-ketolithocholic acid, *7-ketoLCA* 7-ketolithocholic acid, *GLCA* glycolithocholic acid, *THCA* Taurohyocholic acid, *TDCA* Taurodeoxycholic acid, *GUDCA* Glycoursodeoxycholic acid, *GHDCA* Glycohyodeoxycholic acid, *THDCA* Taurohyodeoxycholic acid, *PBA* Primary bile acid, *SBA* Secondary bile acid, *FBA* Free bile acid, *CBA* Conjugated bile acid, *FPBA* Free primary bile acid, *FSBA* Free secondary bile acid, *GPBA* Glycine primary bile acid, *GSBA* Glycine secondary bile acid, *TPBA* Taurine primary bile acid, *TSBA* Taurine secondary bile acid. *SEM* Standard error of means. CON and BAS, without and with supplementing 20 g/d of bile acids, respectively

**Table S4** Effects of supplementing bile acids on fecal bile acid individual and categories on d 21 postpartum in transition dairy cows

| **Items^1^** | **Treatments** | | **SEM** | ***P*-value** |
| --- | --- | --- | --- | --- |
|  | **CON** | **BAS** |  |  |
| BA individual, nmol/kg | |  |  |  |
| TBA | 391324.82 | 1329823.59 | 132803.921 | < 0.01 |
| HCA | 126.87 | 7748.50 | 1343.486 | 0.01 |
| GCA | 7212.98 | 16756.15 | 1967.035 | 0.02 |
| βCA | 23.20 | 192.34 | 23.620 | < 0.01 |
| α-MCA | 18.21 | 302.02 | 38.552 | < 0.01 |
| β-MCA | 62.06 | 1131.67 | 150.246 | < 0.01 |
| GHCA | 6.27 | 663.19 | 100.382 | < 0.01 |
| GCDCA | 803.72 | 1893.07 | 212.183 | 0.01 |
| LCA | 39625.47 | 72006.16 | 5738.917 | < 0.01 |
| UCA | 194.19 | 408.94 | 32.777 | < 0.01 |
| HDCA | 2680.33 | 591046.65 | 81575.162 | < 0.01 |
| UDCA | 55.66 | 311.83 | 34.816 | < 0.01 |
| MDCA | 247.92 | 16331.64 | 2121.549 | < 0.01 |
| ω-MCA | 54.53 | 2255.09 | 298.927 | < 0.01 |
| isoLCA | 7634.45 | 12230.40 | 1022.145 | 0.02 |
| isoHDCA | 304.58 | 63783.36 | 8515.433 | < 0.01 |
| 6-ketoLCA | 410.97 | 56591.20 | 7575.077 | < 0.01 |
| 7-ketoLCA | 149.45 | 309.98 | 39.727 | 0.05 |
| GLCA | 83.36 | 213.39 | 23.093 | < 0.01 |
| THCA | 6.27 | 142.26 | 22.084 | < 0.01 |
| GHDCA | 6.27 | 1346.00 | 209.866 | < 0.01 |
| THDCA | 14.1 | 640.25 | 91.627 | < 0.01 |
| UDCA-3S | 61.65 | 293.21 | 31.800 | < 0.01 |
| BA categories % |  |  |  |  |
| FBA | 92.36 | 96.93 | 0.010 | 0.79 |
| CBA | 7.64 | 3.07 | 0.001 | 0.01 |
| PBA | 6.72 | 2.52 | 0.001 | 0.04 |
| SBA | 93.28 | 97.48 | 0.010 | 0.80 |
| FPBA | 2.27 | 1.44 | 0.000 | 0.27 |
| FSBA | 90.09 | 95.48 | 0.010 | 0.74 |
| GPBA | 0.21 | 0.19 | 0.000 | 0.76 |
| GSBA | 2.58 | 1.76 | 0.000 | 0.10 |
| TPBA | 4.13 | 0.84 | 0.001 | 0.02 |
| TSBA | 0.58 | 0.21 | 0.000 | 0.01 |

^1^ *BAs* Bile acids, *TBA* Total bile acid, *HCA* Hyocholic acid, *GC*A Glycocholic acid, *βCA* 3β-Cholic acid, *α-MCA* α-Muricholic acid, *β-MCA* β-Muricholic acid, *GHCA* Glycohyocholic acid, *GCDCA* Glycochenodeoxycholic acid, *LCA* Lithocholic acid, *UCA* Ursocholic acid, *HDCA* Hyodeoxycholic acid, *UDCA* Ursodeoxycholic acid, *MDCA* Murideoxycholic acid, *ω-MCA* ω-Muricholic acid, *isoLCA* isoallolithocholic acid, *isoHDCA* isohyodeoxycholic acid, *6-ketoLCA* 6-ketolithocholic acid, *7-ketoLCA* 7-ketolithocholic acid, *GLCA* Glycolithocholic acid, *THCA* Taurohyocholic acid, *GHDCA* Glycohyodeoxycholic acid, *THDCA* Taurohyodeoxycholic acid, *UDCA-3S* Ursodeoxycholic acid 3-Sulfate, *PBA* Primary bile acid, *SBA* Secondary bile acid, *FBA* Free bile acid, *CBA* Conjugated bile acid, *FPBA* Free primary bile acid, *FSBA* Free secondary bile acid, *GPBA* Glycine primary bile acid, *GSBA* Glycine secondary bile acid, *TPBA* Taurine primary bile acid, *TSBA* Taurine secondary bile acid, *SEM* Standard error of means. CON and BAS, without and with supplementing 20 g/d of bile acids, respectively
