# Supplementary figures and images for: Effects of supplementing bile acids on the production performance, fatty acid and bile acid composition, and gut microbiota in transition dairy cows
Source: J Anim Sci Biotechnol. 2025 Jun 12;16:83. doi: 10.1186/s40104-025-01207-8 (PMC12160099; doi:10.1186/s40104-025-01207-8)

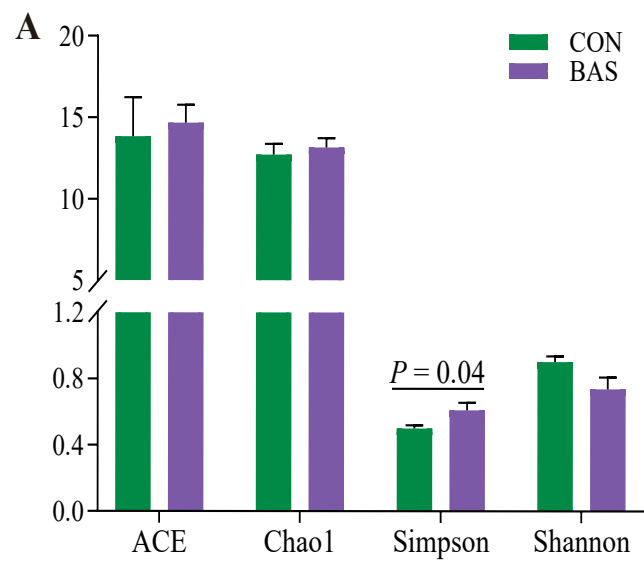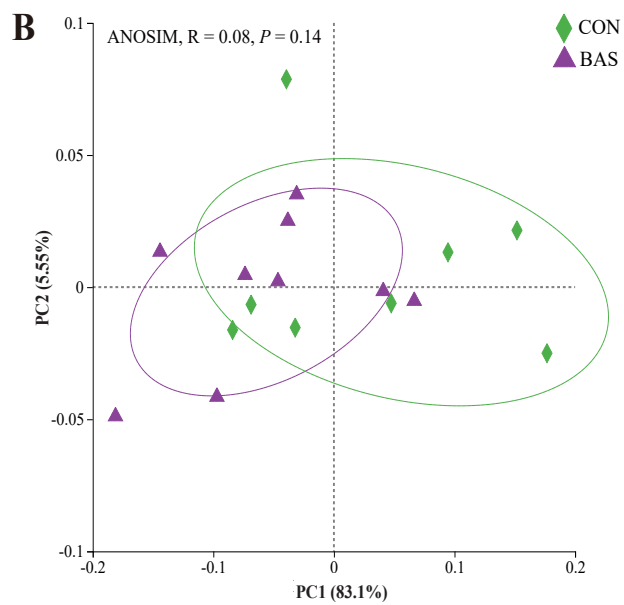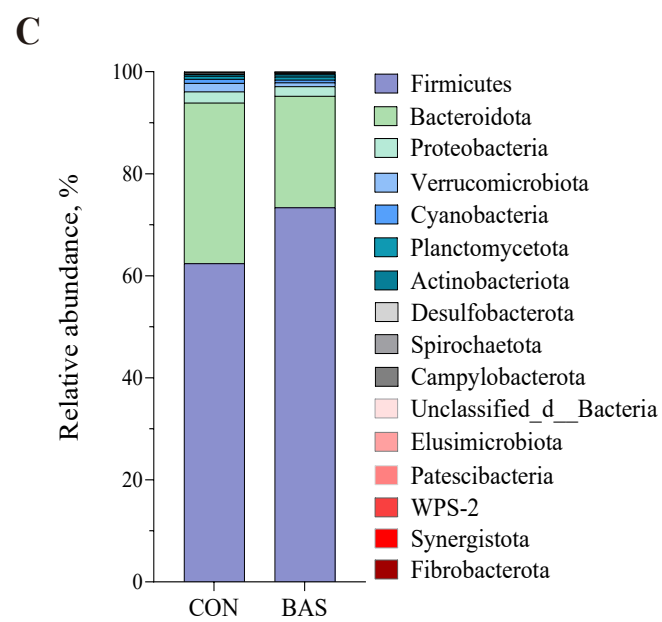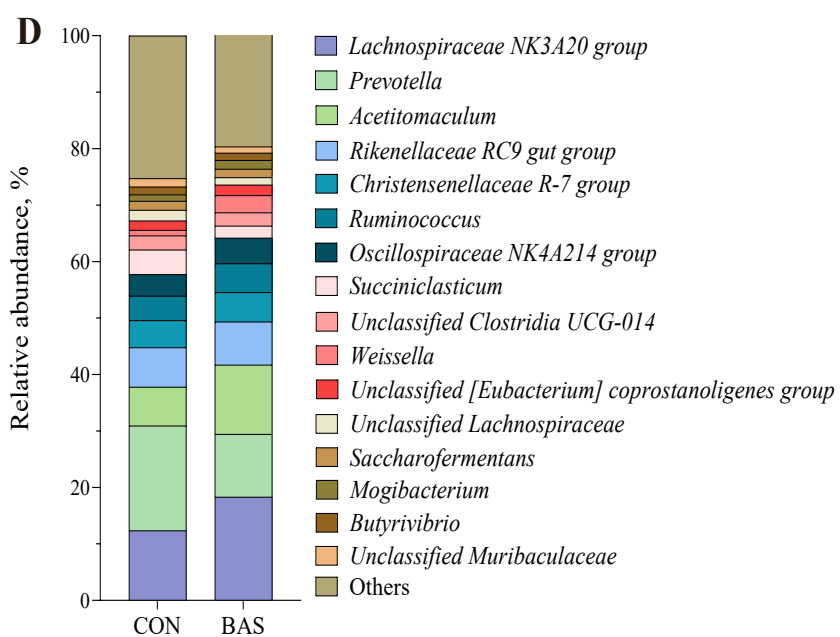

Supplement: Supplementary file 2 — Additional file 2: Fig. S1 Rumen microbial community composition and diversity between CON and BAS groups (n = 9). A The ACE, Chao1, Simpson, and Shannon indices of rumen microbiota were analyzed using the Wilcon rank sum test for differences. B Principal coordinates analysis (PCoA) used Similarity Analysis (ANOSIM) at the phylum level to determine the statistical significance of 999 permutations. C The relative abundance of bacterial phylum in the rumen of the CON and BAS groups. D The relative abundance of bacterial genera in the rumen of the CON and BAS groups. CON and BAS, without and with supplementing 20 g/d of bile acids, respectively. [file 40104_2025_1207_MOESM2_ESM.pdf]
